# 4-(4-Cyano­benzoyl­meth­yl)benzonitrile

**DOI:** 10.1107/S1600536808015079

**Published:** 2008-05-24

**Authors:** Wenxiang Wang, Hong Zhao

**Affiliations:** a Ordered Matter Science Research Center, College of Chemistry and Chemical Engineering, Southeast University, Nanjing 210096, People’s Republic of China

## Abstract

In the title compound, C_16_H_10_N_2_O, the dihedral angle formed by the benzene rings is 84.99 (7)°. The crystal structure is stabilized by inter­molecular C—H⋯N and C—H⋯O hydrogen-bond inter­actions, forming chains running parallel to the *b* axis.

## Related literature

For related literature, see: Arıcı *et al.* (2004 ▶); Radl *et al.* (2000 ▶); Bernstein *et al.* (1995 ▶); Zhao (2008 ▶).
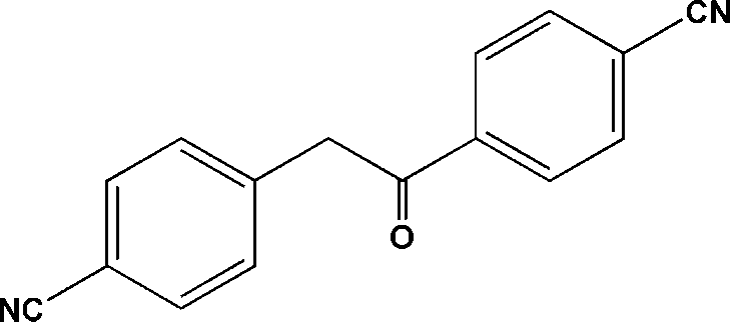


## Experimental

### 

#### Crystal data

C_16_H_10_N_2_O
*M* *_r_* = 246.26
Triclinic, 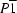

*a* = 7.5217 (15) Å
*b* = 7.9759 (16) Å
*c* = 10.881 (2) Å
α = 96.78 (3)°
β = 93.34 (3)°
γ = 102.10 (3)°
*V* = 631.5 (2) Å^3^
*Z* = 2
Mo *K*α radiation
μ = 0.08 mm^−1^
*T* = 293 (2) K
0.20 × 0.20 × 0.16 mm

#### Data collection

Rigaku Mercury2 diffractometer
Absorption correction: multi-scan (*CrystalClear*; Rigaku/MSC, 2005 ▶) *T*_min_ = 0.964, *T*_max_ = 0.988
6576 measured reflections
2898 independent reflections
1943 reflections with *I* > 2σ(*I*)
*R*_int_ = 0.028

#### Refinement

*R*[*F* ^2^ > 2σ(*F* ^2^)] = 0.059
*wR*(*F* ^2^) = 0.157
*S* = 1.04
2898 reflections
173 parameters
H-atom parameters constrained
Δρ_max_ = 0.25 e Å^−3^
Δρ_min_ = −0.21 e Å^−3^

### 

Data collection: *CrystalClear* (Rigaku/MSC, 2005 ▶); cell refinement: *CrystalClear*; data reduction: *CrystalClear*; program(s) used to solve structure: *SHELXS97* (Sheldrick, 2008 ▶); program(s) used to refine structure: *SHELXL97* (Sheldrick, 2008 ▶); molecular graphics: *SHELXTL* (Sheldrick, 2008 ▶); software used to prepare material for publication: *SHELXTL*.

## Supplementary Material

Crystal structure: contains datablocks I, global. DOI: 10.1107/S1600536808015079/rz2215sup1.cif

Structure factors: contains datablocks I. DOI: 10.1107/S1600536808015079/rz2215Isup2.hkl

Additional supplementary materials:  crystallographic information; 3D view; checkCIF report

## Figures and Tables

**Table 1:** Hydrogen-bond geometry (Å, °)

*D*—H⋯*A*	*D*—H	H⋯*A*	*D*⋯*A*	*D*—H⋯*A*
C3—H3⋯N1^i^	0.93	2.62	3.486 (3)	154
C12—H12⋯O1^ii^	0.93	2.42	3.268 (2)	152

Symmetry codes: (i) 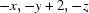; (ii) 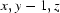.

**Fig. 1.**
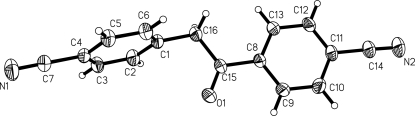
The molecular structure of the title compound with the atom-numbering scheme. Displacement ellipsoids were drawn at the 30% probability level

## supplementary crystallographic information

### Comment

Benzonitriles and their derivatives are important starting materials in the synthesis of some heterocyclic molecules (Radl *et al.*, 2000; Arıcı *et al.*, 2004). As part of our ongoing study on benzonitrile derivatives (Zhao, 2008), the crystal structure of one such derivatives is reported here.

The molecular structure of the title compound (Fig. 1) shows normal bond lengths and angles. The C≡N triple bond and C=O double bond lengths are 1.142 (2) and and 1.193 (2) Å, respectively. The benzene ring are oriented nearly perpendicular to each other, the dihedral angle they form being 84.99 (7)°. In the crystal structure, centrosymmetrically-related molecules are linked into dimeric units by intermolecular C—H···N hydrogen bonds (Table 1) forming ten-membered rings of graph-set R^2^_2_(10) (Berstein *et al.*, 1995). These dimers are further connected by intermolecular C—H···O hydrogen interactions to form chains running parallel to the *b* axis.

### Experimental

To a solution of  sodium cyanide (2 g) in water (18 ml) was added 4-formylbenzonitrile (2.62 g). The mixture was stirred for 15 min at room temperature, then a saturated sodium hydrosulfite solution (15 ml) was added dropwise. The resulting mixture was stirred at 293K until a yellow solid was obtained. The solid was filtered and recrystallized from a mixture of methanol (18 ml) and DMF (6 ml), to give crystals of the title compound suitable for X-ray diffraction analysis on slow evaporation of the solvents.

### Refinement

All hydrogen atoms were placed at calculated positions and refined using the riding model approximation, with C—H = 0.93-0.97 Å and with *U*_iso_(H) = 1.2 *U*_eq_(C).

### Crystal data

**Table:**

C_16_H_10_N_2_O	*Z* = 2
*M**_r_* = 246.26	*F*_000_ = 256
Triclinic, *P*1	*D*_x_ = 1.295 Mg m^−^^3^
Hall symbol: -P 1	Mo *K*α radiation λ = 0.71073 Å
*a* = 7.5217 (15) Å	Cell parameters from 2232 reflections
*b* = 7.9759 (16) Å	θ = 3.0–27.4º
*c* = 10.881 (2) Å	µ = 0.08 mm^−^^1^
α = 96.78 (3)º	*T* = 293 (2) K
β = 93.34 (3)º	Block, yellow
γ = 102.10 (3)º	0.20 × 0.20 × 0.16 mm
*V* = 631.5 (2) Å^3^	

### Data collection

**Table:**

Rigaku Mercury2 diffractometer	2898 independent reflections
Radiation source: fine-focus sealed tube	1943 reflections with *I* > 2σ(*I*)
Monochromator: graphite	*R*_int_ = 0.028
Detector resolution: 13.6612 pixels mm^-1^	θ_max_ = 27.5º
*T* = 293(2) K	θ_min_ = 3.0º
CCD_Profile_fitting scans	*h* = −9→9
Absorption correction: Multi-scan(CrystalClear; Rigaku/MSC, 2005)	*k* = −10→10
*T*_min_ = 0.964, *T*_max_ = 0.988	*l* = −14→14
6576 measured reflections	

### Refinement

**Table:**

Refinement on *F*^2^	Hydrogen site location: inferred from neighbouring sites
Least-squares matrix: full	H-atom parameters constrained
*R*[*F*^2^ > 2σ(*F*^2^)] = 0.059	*w* = 1/[σ^2^(*F*_o_^2^) + (0.0717*P*)^2^ + 0.1009*P*] where *P* = (*F*_o_^2^ + 2*F*_c_^2^)/3
*wR*(*F*^2^) = 0.157	(Δ/σ)_max_ = 0.002
*S* = 1.04	Δρ_max_ = 0.25 e Å^−^^3^
2898 reflections	Δρ_min_ = −0.21 e Å^−^^3^
173 parameters	Extinction correction: SHELXL97 (Sheldrick, 2008), Fc^*^=kFc[1+0.001xFc^2^λ^3^/sin(2θ)]^-1/4^
Primary atom site location: structure-invariant direct methods	Extinction coefficient: 0.168 (18)
Secondary atom site location: difference Fourier map	

### Special details

**Table:**

Geometry. All e.s.d.'s (except the e.s.d. in the dihedral angle between two l.s. planes) are estimated using the full covariance matrix. The cell e.s.d.'s are taken into account individually in the estimation of e.s.d.'s in distances, angles and torsion angles; correlations between e.s.d.'s in cell parameters are only used when they are defined by crystal symmetry. An approximate (isotropic) treatment of cell e.s.d.'s is used for estimating e.s.d.'s involving l.s. planes.
Refinement. Refinement of *F*^2^ against ALL reflections. The weighted *R*-factor *wR* and goodness of fit *S* are based on *F*^2^, conventional *R*-factors *R* are based on *F*, with *F* set to zero for negative *F*^2^. The threshold expression of *F*^2^ > σ(*F*^2^) is used only for calculating *R*-factors(gt) *etc*. and is not relevant to the choice of reflections for refinement. *R*-factors based on *F*^2^ are statistically about twice as large as those based on *F*, and *R*- factors based on ALL data will be even larger.

### Fractional atomic coordinates and isotropic or equivalent isotropic displacement parameters (Å^2^)

**Table:**

	*x*	*y*	*z*	*U*_iso_*/*U*_eq_	
C1	0.1774 (2)	0.4661 (2)	0.18751 (15)	0.0468 (4)	
C2	0.0347 (3)	0.5506 (2)	0.18255 (17)	0.0546 (5)	
H2	−0.0715	0.5081	0.2189	0.066*	
C3	0.0475 (3)	0.6970 (2)	0.12455 (17)	0.0532 (5)	
H3	−0.0493	0.7528	0.1218	0.064*	
C4	0.2055 (2)	0.7599 (2)	0.07065 (15)	0.0468 (4)	
C5	0.3487 (3)	0.6762 (2)	0.07410 (17)	0.0550 (5)	
H5	0.4545	0.7181	0.0371	0.066*	
C6	0.3339 (3)	0.5301 (2)	0.13273 (17)	0.0550 (5)	
H6	0.4306	0.4742	0.1353	0.066*	
C7	0.2217 (3)	0.9131 (2)	0.00999 (17)	0.0558 (5)	
C8	0.2389 (2)	0.2017 (2)	0.46012 (15)	0.0457 (4)	
C9	0.2979 (3)	0.2430 (2)	0.58529 (16)	0.0580 (5)	
H9	0.3178	0.3571	0.6226	0.070*	
C10	0.3274 (3)	0.1162 (2)	0.65476 (17)	0.0599 (5)	
H10	0.3668	0.1447	0.7387	0.072*	
C11	0.2980 (2)	−0.0536 (2)	0.59929 (16)	0.0476 (4)	
C12	0.2369 (2)	−0.0974 (2)	0.47534 (17)	0.0521 (5)	
H12	0.2158	−0.2118	0.4384	0.062*	
C13	0.2074 (3)	0.0309 (2)	0.40669 (16)	0.0508 (5)	
H13	0.1656	0.0018	0.3232	0.061*	
C14	0.3367 (3)	−0.1839 (2)	0.67244 (18)	0.0560 (5)	
C15	0.2138 (3)	0.3455 (2)	0.38867 (17)	0.0589 (5)	
N1	0.2355 (3)	1.0349 (2)	−0.03745 (18)	0.0757 (5)	
N2	0.3690 (3)	−0.2834 (2)	0.73238 (17)	0.0742 (6)	
O1	0.2335 (4)	0.48933 (19)	0.44054 (14)	0.1312 (10)	
C16	0.1648 (3)	0.3057 (2)	0.25106 (16)	0.0529 (5)	
H16A	0.0413	0.2365	0.2358	0.063*	
H16B	0.2458	0.2374	0.2147	0.063*	

### Atomic displacement parameters (Å^2^)

**Table:**

	*U*^11^	*U*^22^	*U*^33^	*U*^12^	*U*^13^	*U*^23^
C1	0.0585 (11)	0.0387 (8)	0.0433 (9)	0.0106 (8)	−0.0022 (8)	0.0092 (7)
C2	0.0563 (11)	0.0512 (10)	0.0595 (11)	0.0119 (9)	0.0080 (9)	0.0189 (9)
C3	0.0581 (11)	0.0491 (10)	0.0585 (11)	0.0204 (8)	0.0042 (9)	0.0166 (8)
C4	0.0600 (11)	0.0400 (8)	0.0405 (8)	0.0105 (8)	−0.0016 (8)	0.0090 (7)
C5	0.0575 (11)	0.0531 (10)	0.0572 (11)	0.0126 (9)	0.0064 (9)	0.0171 (9)
C6	0.0573 (11)	0.0531 (10)	0.0592 (11)	0.0196 (9)	0.0014 (9)	0.0142 (9)
C7	0.0694 (13)	0.0504 (10)	0.0516 (10)	0.0168 (9)	0.0060 (9)	0.0153 (8)
C8	0.0579 (10)	0.0367 (8)	0.0456 (9)	0.0140 (7)	0.0060 (8)	0.0104 (7)
C9	0.0864 (14)	0.0370 (9)	0.0500 (10)	0.0134 (9)	0.0019 (10)	0.0055 (8)
C10	0.0798 (14)	0.0507 (10)	0.0478 (10)	0.0099 (10)	−0.0036 (9)	0.0133 (8)
C11	0.0474 (9)	0.0431 (9)	0.0569 (10)	0.0109 (7)	0.0092 (8)	0.0212 (8)
C12	0.0656 (12)	0.0330 (8)	0.0596 (11)	0.0121 (8)	0.0076 (9)	0.0106 (8)
C13	0.0668 (11)	0.0378 (9)	0.0486 (10)	0.0132 (8)	0.0022 (8)	0.0073 (7)
C14	0.0566 (11)	0.0512 (10)	0.0661 (12)	0.0144 (9)	0.0103 (9)	0.0243 (9)
C15	0.0930 (15)	0.0367 (9)	0.0514 (10)	0.0225 (9)	0.0028 (10)	0.0108 (8)
N1	0.0880 (13)	0.0672 (11)	0.0853 (13)	0.0269 (10)	0.0212 (10)	0.0399 (10)
N2	0.0837 (13)	0.0672 (11)	0.0843 (13)	0.0263 (10)	0.0116 (10)	0.0410 (10)
O1	0.296 (3)	0.0429 (8)	0.0603 (10)	0.0630 (13)	−0.0189 (13)	0.0018 (7)
C16	0.0680 (12)	0.0387 (9)	0.0527 (10)	0.0126 (8)	−0.0010 (9)	0.0108 (8)

### Geometric parameters (Å, °)

**Table:**

C1—C6	1.380 (3)	C8—C15	1.496 (2)
C1—C2	1.383 (2)	C9—C10	1.377 (2)
C1—C16	1.513 (2)	C9—H9	0.9300
C2—C3	1.381 (2)	C10—C11	1.384 (3)
C2—H2	0.9300	C10—H10	0.9300
C3—C4	1.381 (3)	C11—C12	1.379 (2)
C3—H3	0.9300	C11—C14	1.446 (2)
C4—C5	1.382 (3)	C12—C13	1.382 (2)
C4—C7	1.442 (2)	C12—H12	0.9300
C5—C6	1.381 (2)	C13—H13	0.9300
C5—H5	0.9300	C14—N2	1.140 (2)
C6—H6	0.9300	C15—O1	1.193 (2)
C7—N1	1.142 (2)	C15—C16	1.501 (3)
C8—C13	1.383 (2)	C16—H16A	0.9700
C8—C9	1.387 (2)	C16—H16B	0.9700
			
C6—C1—C2	118.89 (15)	C8—C9—H9	119.7
C6—C1—C16	119.65 (16)	C9—C10—C11	119.80 (17)
C2—C1—C16	121.46 (16)	C9—C10—H10	120.1
C3—C2—C1	121.07 (17)	C11—C10—H10	120.1
C3—C2—H2	119.5	C12—C11—C10	120.45 (15)
C1—C2—H2	119.5	C12—C11—C14	120.50 (16)
C2—C3—C4	119.32 (17)	C10—C11—C14	119.03 (17)
C2—C3—H3	120.3	C11—C12—C13	119.21 (16)
C4—C3—H3	120.3	C11—C12—H12	120.4
C3—C4—C5	120.28 (15)	C13—C12—H12	120.4
C3—C4—C7	120.13 (16)	C12—C13—C8	121.10 (17)
C5—C4—C7	119.59 (16)	C12—C13—H13	119.5
C6—C5—C4	119.70 (17)	C8—C13—H13	119.5
C6—C5—H5	120.2	N2—C14—C11	178.3 (2)
C4—C5—H5	120.2	O1—C15—C8	120.30 (17)
C1—C6—C5	120.74 (17)	O1—C15—C16	120.78 (16)
C1—C6—H6	119.6	C8—C15—C16	118.92 (15)
C5—C6—H6	119.6	C15—C16—C1	113.15 (14)
N1—C7—C4	179.5 (2)	C15—C16—H16A	108.9
C13—C8—C9	118.91 (15)	C1—C16—H16A	108.9
C13—C8—C15	122.97 (16)	C15—C16—H16B	108.9
C9—C8—C15	118.12 (15)	C1—C16—H16B	108.9
C10—C9—C8	120.52 (16)	H16A—C16—H16B	107.8
C10—C9—H9	119.7		

### Hydrogen-bond geometry (Å, °)

**Table:**

*D*—H···*A*	*D*—H	H···*A*	*D*···*A*	*D*—H···*A*
C3—H3···N1^i^	0.93	2.62	3.486 (3)	154
C12—H12···O1^ii^	0.93	2.42	3.268 (2)	152

Symmetry codes: (i) −*x*, −*y*+2, −*z*; (ii) *x*, *y*−1, *z*.

## References

[bb1] Arıcı, C., Ülkü, D., Kırılmış, C., Koca, M. & Ahmedzade, M. (2004). *Acta Cryst.* E**60**, o1211–o1212.

[bb2] Bernstein, J., Davis, R. E., Shimoni, L. & Chang, N.-L. (1995). *Angew. Chem. Int. Ed. Engl* **34**, 1555–1573.

[bb3] Radl, S., Hezky, P., Konvicka, P. & Krejgi, J. (2000). *Collect. Czech. Chem. Commun.* **65**, 1093–1108.

[bb4] Rigaku/MSC (2005). *CrystalClear* and *CrystalStructure* Rigaku/MSC, The Woodlands, Texas, USA.

[bb5] Sheldrick, G. M. (2008). *Acta Cryst.* A**64**, 112–122. 10.1107/S0108767307043930 18156677

[bb6] Zhao, Y.-Y. (2008). *Acta Cryst.* E**64**, o761. 10.1107/S1600536808007629 PMC2960981 21202150

